# Potential of Proliferative Markers in Pancreatic Cancer Management: A Systematic Review

**DOI:** 10.1002/hsr2.70412

**Published:** 2025-03-05

**Authors:** Aryan Salahi‐Niri, Paniz Zarand, Negar Mansouri, Parvaneh Rastgou, Omid Yazdani, Romina Esbati, Fatemeh Shojaeian, Behnaz Jahanbin, Zhaleh Mohsenifar, Hamid Asadzadeh Aghdaei, Farid Azmoudeh Ardalan, Seyed Amir Ahmad Safavi‐Naini

**Affiliations:** ^1^ Research, Institute for Gastroenterology and Liver Diseases Shahid Beheshti University of Medical Sciences Tehran Iran; ^2^ School of Medicine Tabriz University of Medical Sciences Tabriz Iran; ^3^ Sidney Kimmel Comprehensive Cancer Research Center Johns Hopkins School of Medicine Baltimore Maryland USA; ^4^ Cancer Institute, Pathology Department, Imam Khomeini Hospital Complex Tehran University of Medical Sciences Tehran Iran; ^5^ Department of Pathology, Ayatollah Taleghani Educational Hospital, Faculty of Medicine Shahid Beheshti University of Medical Sciences Tehran Iran; ^6^ Pathology and Laboratory Medicine Department, Imam Khomeini Hospital Complex Tehran University of Medical Sciences Tehran Iran

**Keywords:** Cyclin D1, Ki‐67, pancreatic cancer, PCNA, proliferative markers

## Abstract

**Background and Aims:**

Pancreatic cancer is an aggressive malignancy with poor prognosis and limited treatment options. Chemotherapy remains a primary therapeutic approach, but patient responses vary significantly, emphasizing the need for reliable biomarkers. This review explores the potential role of proliferative markers, including Ki‐67, PCNA, Cyclin D1, and PHH3, as predictive and prognostic indicators in pancreatic cancer management, aiming to enhance personalized treatment strategies.

**Methods:**

We conducted a narrative review by searching Scopus, PubMed, and Google Scholar for studies focusing on Ki‐67, PCNA, Cyclin D1, and PHH3 in relation to pancreatic cancer and chemotherapy. The literature was reviewed to evaluate the role of these markers in predicting chemotherapy response, tumor progression, and overall patient survival.

**Results:**

The review highlights the clinical significance of these markers. Ki‐67 and PCNA are associated with cell proliferation, while Cyclin D1 regulates cell cycle progression and PHH3 is linked to mitotic activity. High expression levels of these markers often correlate with increased tumor aggressiveness and poorer patient outcomes. Moreover, they show promise in predicting chemotherapy response, which can inform tailored therapeutic strategies. However, challenges remain, including standardization of detection methods and determination of optimal cutoff values.

**Conclusion:**

Proliferative markers such as Ki‐67, PCNA, Cyclin D1, and PHH3 hold potential as predictive and prognostic tools in pancreatic cancer management. Their integration into clinical practice could improve the accuracy of treatment decisions and enhance patient outcomes. Further research and validation are necessary to overcome existing challenges and optimize their application in personalized oncology.

## 1 Introduction

Pancreatic cancer is a highly aggressive malignancy with a dismal survival rate, primarily affecting men and older adults between 60 and 85 years old. It accounts for 7% of all cancer deaths and is the eighth most common cancer in women and the tenth in men [1]. In the United States, it is estimated that 62,210 adults (32,970 men and 29,240 women) will be diagnosed with pancreatic cancer in 2022, with an increasing incidence among younger patients. Due to the lack of early symptoms and rapid invasion of surrounding tissues and organs, pancreatic cancer is among the most lethal of all cancers [2, 3].

Early diagnosis and treatment planning are crucial for improving detection and survival rates of pancreatic cancer [4]. Proliferative markers are now being used as diagnostic and treatment methods in conjunction with surgical and medical therapies such as robotic surgery and neoadjuvant chemoradiotherapy [5]. However, the outcomes of these treatments have only demonstrated modest improvement, highlighting the urgent need for more effective therapies [6]. Molecular cancer biomarkers refer to any measurable molecular indicator that can predict the risk, occurrence, or outcome of cancer in patients [7]. These biomarkers can include germline or somatic genetic variants, transcriptional changes, and proteomic signatures, all based on biomolecules such as nucleic acids and proteins [8]. Proteins are the primary biological molecules that execute cellular functions and are currently the only approved biomarkers for cancer diagnosis and monitoring [9].

Biomarkers have broad clinical applications, including the assessment of cancer risk, early‐stage cancer screening and detection, precise cancer diagnosis, prognosis prediction, anticipation of therapy response, and monitoring cancer progression [10]. They can help optimize decision‐making in clinical practice [11]. Precision oncology requires the identification of specific cancer genetic mutations that are functional only in patients with these mutations, and biomarkers are essential tools for identifying these subsets of patients [12].

The development of new biomarkers that are more sensitive and specific, with a higher positive predictive value, is necessary to advance the field of cancer biomarkers. This improvement is crucial for developing new targeted therapies that can provide more effective treatment options for cancer patients [7]. For example, Cyclin D1, Ki‐67, phosphorylated histone H3 (PHH3), and proliferating cell nuclear antigen (PCNA) are standard proliferation markers used to assess the growth fraction of a cell population [13]. These proteins have been identified in various malignant tumors, including pancreatic cancer, and serve as potential diagnostic tools [14].

This narrative review explores the potential of proliferative markers, namely Ki‐67, PCNA, PHH3, and Cyclin D1, as predictive and prognostic tools in pancreatic cancer chemotherapy. We studied their roles in chemotherapy response, overall survival, and tumor progression, while addressing critical issues like standardization and clinical integration. This review offers a comprehensive perspective on how these markers may revolutionize personalized pancreatic cancer management, underscoring their significance in improving treatment outcomes.

## 2 Search Method

We conducted a search through Scopus, PubMed, and Google Scholar on March 23, 2023, to find relevant studies. The search strategy in each database included the following terms: (“KI‐67” OR “Cyclin D1” OR “PCNA” OR “PHH3” OR “proliferative markers” OR “proliferation factors”) AND (“Pancreas cancer” OR “Pancreas carcinoma”) AND (“Chemo*”). Studies on chemotherapy were selected by two reviewers and grouped into subheadings of “Value of markers before chemotherapy” and “The effect of chemotherapy on markers.” An update of the search was made on December 24, 2023.

We reviewed clinical trials, observational and retrospective studies examining proliferative markers (Ki‐67, PCNA, Cyclin D1, PHH3) in pancreatic cancer, focusing on their prognostic and predictive value. We included English‐language, peer‐reviewed studies published since 2000. Studies were excluded if they addressed other cancers, lacked clear prognostic/predictive data, were case reports or reviews, or had inadequate sample sizes or methodology.

## 3 Overview of Markers in Pancreatic Cancer Care

The diagnosis and prognosis of pancreatic cancer heavily depend on identifying specific markers due to the illness's complexity and multiple molecular abnormalities [15]. In addition to proliferative markers such as Cyclin D1 and Ki‐67, genetic markers like KRAS mutations and changes in tumor suppressor genes like TP53 provide insights into the genetic landscape of the disease [16, 17]. Proteomic profiles and proteins linked to carbohydrate metabolism (e.g., CA 242) add to the variety of diagnostic tools [18]. While these markers collectively enhance our ability to detect and characterize pancreatic cancer, the importance of proliferative markers cannot be overstated [19]. Proliferative markers not only signify tumor aggressiveness but also guide clinicians in tailoring treatment strategies, underscoring their pivotal role in managing pancreatic cancer [20, 21].

Different proliferative markers are essential for understanding tumor behavior and guiding therapeutic choices. The nuclear protein Ki‐67, linked to cell division, is often measured in pancreatic cancer samples. High levels of Ki‐67 expression correlate with increased tumor aggressiveness, helping doctors forecast disease progression [22]. Proliferating cell nuclear antigen, or PCNA, is another important marker that is involved in DNA replication and repair. Increased PCNA levels provide important information about the biological features of pancreatic tumors by indicating active cell proliferation [23].

Cyclin D1, an important cell cycle regulator, has become a notable marker. Its upregulation in tissues from pancreatic cancer is a sign of an aggressive disease and is linked to enhanced cell proliferation [24]. Phosphohistone H3 (PHH3), a mitotic marker that uniquely identifies cells in the late G2 and M phases, is another intriguing marker. Increased PHH3 levels indicate increased mitotic activity in pancreatic cancers, which can help determine how aggressive a tumor is [25]. By incorporating these markers into prognostic and diagnostic assessments, pancreatic cancer treatment can be more precisely targeted [26]. Tracking these indicators' changes throughout the course of treatment allows for more customized therapeutic approaches and a deeper understanding of the molecular features of the tumor, both of which improve patient outcomes [27]. Table 1 provides a summary of the use of Ki‐67, PCNA, PHH3, and Cyclin D1 proteins in assessing the growth fraction of a cell population.

**Table 1:** Description and function of proliferative markers in pancreas cancer care.

Immunohistochemistry Markers		
Marker	Description	Function
PHH3	A particular marker that pathologists employ to recognize cells actively undergoing mitosis. Histone H3, a protein involved in the structural structure of chromatin during cell division, is known as PHH3. PHH3 is created when histone H3 is phosphorylated at the serine 10 residue, which only happens in the late G2 and M phases of the cell cycle. PHH3 is a useful immunohistochemistry marker for identifying and measuring cells actively undergoing mitosis in tissue samples [28].	PHH3's main job as a mitotic marker is to fulfill this task. As cells advance through the cell cycle and into the latter phases of the G2 and M phases, PHH3 is more prevalent. Researchers and pathologists can precisely identify and count mitotic cells using immunohistochemical detection of PHH3, offering insights into the proliferative activity of tissues [29]. Regarding pancreatic cancer research, PHH3 is essential for evaluating the mitotic index, which is a measurement of the percentage of cells in a tumor that is actively dividing. This information helps understand the aggressiveness of pancreatic tumors and guides treatment options and prognoses [30].
Cyclin D1	Cyclin D1 is one of the proto‐oncogenes frequently amplified in cancer. The identification of its proto‐oncogenic role originated from studies on gene amplification, revealing its association with a chromosome inversion in parathyroid adenoma (PRAD1). It was also isolated from a human glioblastoma library using yeast genetic selection in G1‐cyclin depleted cells [31].	Cyclin D1 is a crucial regulator of G1 to S phase progression in various cell types. It forms active complexes with cyclin‐dependent kinase 4 and 6 (CDK4/6), promoting cell cycle progression by phosphorylating and inactivating the retinoblastoma protein. Recent studies have revealed its functions as a transcriptional modulator by regulating the activity of several transcription factors and histone deacetylases, providing a new perspective on its role in cancer development and progression [31].
PCNA	PCNA is a human sliding clamp protein that plays a crucial role in DNA replication and repair, making it an ideal therapeutic target for proliferative diseases such as cancer [32].	As a component of the replication and repair machinery, PCNA is indispensable in nucleic acid metabolism. Its toroidal shape enables the protein to encircle DNA and slide bidirectionally along the duplex, facilitating rapid and processive DNA synthesis. PCNA also functions as a tether, binding the polymerase catalytic unit to the DNA template, ensuring efficient and accurate DNA replication and repair [32].
Ki‐67	The Ki‐67 antigen was originally identified by Gerdes and colleagues in the early 1980s, by the use of mouse monoclonal antibody against a nuclear antigen from a Hodgkin's lymphoma‐derived cell line. This nonhistone protein was named after the researchers' location, Ki for Kiel University, Germany, with the 67 label referring to the clone number on the 96‐well plate [33].	For decades, the Ki‐67 protein has served as a widely employed marker for assessing the proliferation of human tumor cells. Ki‐67 has roles in both interphase and mitotic cells, and its cellular distribution dramatically changes during cell cycle progression. For example, during interphase, Ki‐67 is required for normal cellular distribution of heterochromatin antigens and for the nucleolar association of heterochromatin [33].

**Figure 1:**
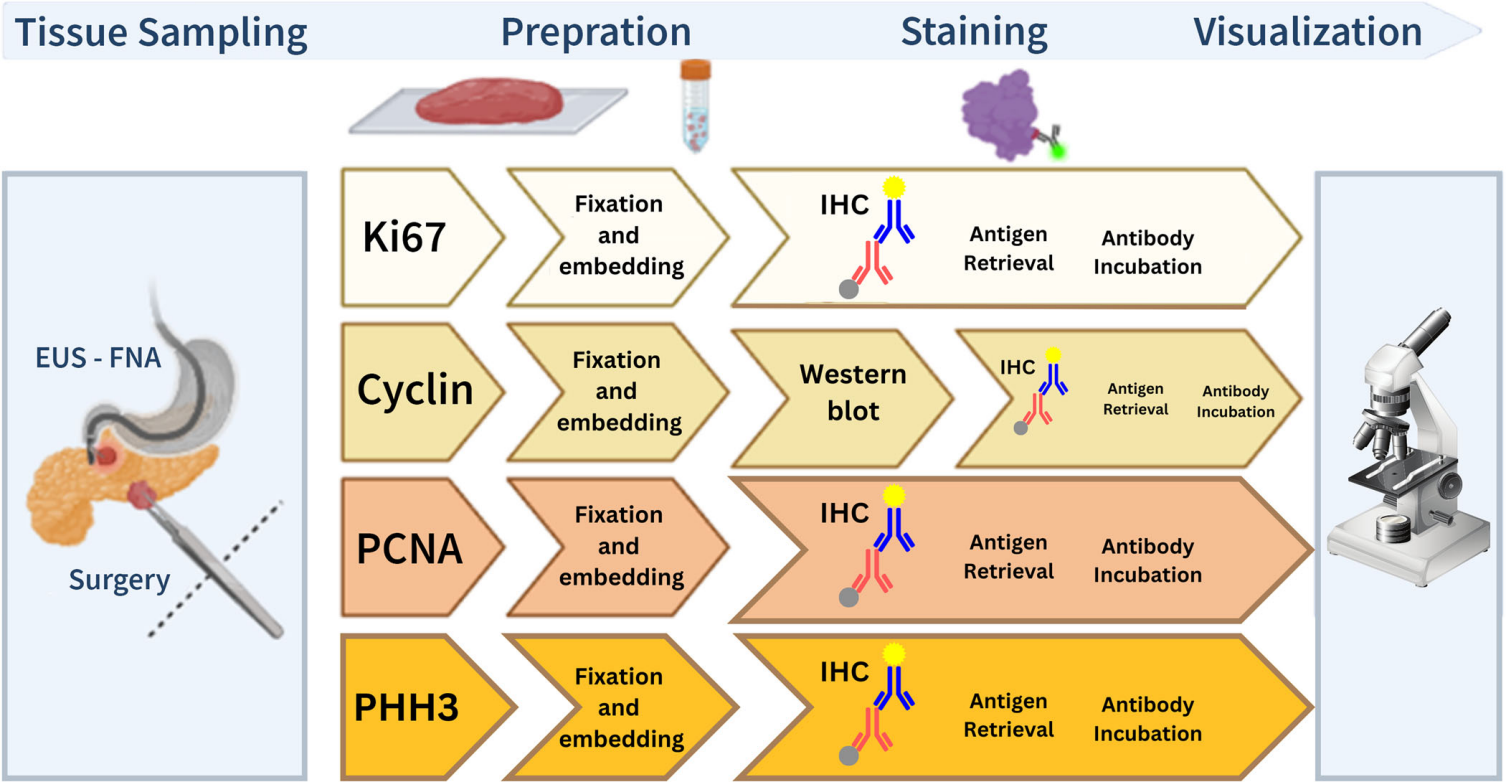
Preparation and staining procedure of four markers: Ki67, Cyclin, PCNA, and PHH3.

Since Ki‐67, Cyclin D1, PHH3, and PCNA are key proliferative markers in pancreatic cancer, our review focuses extensively on them. Nuclear protein Ki‐67, which is linked to cell proliferation, offers important information on tumor aggressiveness [34]. Cyclin D1, a crucial cell cycle regulator, is overexpressed in pancreatic cancer tissues and signals increased cell proliferation [35]. PHH3, a particular marker for cells in the late G2 and M phases, is included to help determine mitotic activity and comprehend the tumor's dynamic character [36]. Additionally, a critical marker for actively dividing cells is PCNA, which is involved in DNA replication and repair. Figure 1 outlines the detailed preparation and staining procedure for these biomarkers. By focusing on these indicators, we hope to draw attention to each one's unique contributions to understanding the proliferative features of pancreatic cancers [37].

### 3.1 Role of Markers in Clinical Management

The use of proliferative markers such as Ki‐67, Cyclin D1, PHH3, and PCNA has garnered increasing attention for their potential as prognostic and predictive tools in cancer treatment, including pancreatic cancer [38]. Despite the promising findings, translating these markers into routine clinical practice presents several challenges [39]. Although many studies have shown a correlation between these markers and cancer aggressiveness, integrating them into therapeutic decision‐making remains complex [35].

Ki‐67's role as a proliferation marker shows mixed results in pancreatic cancer. While studies by Klein et al. [40] and Zińczuk et al. [41] demonstrate correlation between high Ki‐67 levels and decreased survival, other research shows inconsistent findings. These variations may stem from differences in methodology, varying cut‐off values, and tumor heterogeneity [33, 40]. Despite Ki‐67's prognostic potential, its clinical utility remains limited by lack of standardization and absence of treatment protocol guidelines, necessitating further prospective validation [33].

Cyclin D1, frequently overexpressed in pancreatic cancers, correlates with increased proliferation and apoptosis resistance [40]. While its overexpression suggests aggressive tumor behavior and therapy resistance, evidence supporting its use in guiding pancreatic cancer treatment remains limited. Unlike in breast cancer, where Cyclin D1 influences neoadjuvant chemotherapy choices, pancreatic cancer lacks clinical trial data supporting Cyclin D1‐based treatment decisions. Additional prospective trials are needed to validate its predictive value [41].

Overall, Ki‐67, Cyclin D1, PHH3, and PCNA biomarkers can potentially improve pancreatic cancer clinical management by providing valuable information about disease progression and treatment response [9]. However, further research is needed to fully understand their role and determine the most effective ways to incorporate this information into clinical practice [42]. Understanding the predictive value of proliferation activity markers requires comprehending their relationship with traditional prognostic factors such as tumor grade, stage, lymph node metastasis, lymphovascular invasion, and perineural invasion [43]. Proliferation activity markers include Ki‐67, PCNA, PHH3, and Cyclin D1. Research shows that these markers frequently correlate with tumor grade, indicating the degree of aggressiveness and propensity for rapid growth [44]. Research is ongoing to determine if these markers are better than conventional prognostic variables. Some studies suggest that proliferative markers may provide more information on tumor behavior than traditional parameters alone due to their direct role in cell cycle regulation [45].

Research results have been inconsistent regarding their status as independent risk factors for tumor development and poor response to treatment. Although aggressive tumor features are linked to high expression of Ki‐67, PCNA, PHH3, and cyclin D1, their independence as prognostic markers frequently depends on the particular context and patient group [46]. Determining these markers' actual predictive usefulness requires an understanding of how they interact with known prognostic variables. Moreover, current studies seek to clarify if these proliferative markers can function as independent predictors of chemo response, offering important insights for customizing therapeutic approaches [47].

Incorporating biomarkers such as Ki‐67, cyclinD1, PHH3, and PCNA into clinical practice for the management of pancreatic cancer poses several challenges [48]. The clinical implementation of proliferative markers faces significant challenges, primarily due to limited prospective validation studies. While retrospective analyses link these markers to tumor aggressiveness, evidence supporting their use in treatment decisions remains scarce. The requirement for invasive tissue sampling further restricts their widespread adoption [41, 49].

Clinical integration requires standardized assessment protocols, including unified expression cut‐off values, understanding of molecular pathway interactions, and correlation with treatment responses. Prospective trials evaluating their role in treatment decisions are crucial for routine clinical implementation [33].

Ki‐67, PCNA, and Cyclin D1 offer valuable insights into pancreatic cancer biology and PanIN progression [50]. Studies by Klein et al. and Zińczuk et al. show Ki‐67 indices increase with PanIN severity, while PCNA and Cyclin D1 overexpression correlate with advanced PanIN and invasive adenocarcinoma, suggesting utility in identifying high‐risk lesions requiring aggressive intervention [41]. Table 2 sets out clinical significance of proliferative markers in pancreatic cancer.

**Table 2:** Clinical significance of proliferative markers in pancreatic cancer.

Marker	Study	Normal ducts (%)	PanIN‐1 (%)	PanIN‐2 (%)	PanIN‐3 (%)
Ki‐67	Klein et al.	0.41	0.69–2.33	14.08	22.01
Ki‐67	Zińczuk et al.	1.2	4.0	7.5	14.2
PCNA	Zińczuk et al.	88.9	93.8	96.5	97.6
Cyclin D1	Zińczuk et al.	0.7	12.9	35.7	59.5

### 3.2 Multi‐Marker Panels in Pancreatic Cancer

Creating multi‐marker panels including several biomarkers, such as PCNA, cyclin D1, and Ki‐67, may be a better way to predict patient outcomes and direct therapy choices for pancreatic cancer [49, 51]. These multi‐marker panels have the potential to increase prognostic prediction accuracy and offer a more thorough understanding of the molecular profile of pancreatic cancer [52]. Some of the most promising multi‐marker panels include:

1. Panel includes Ki‐67, cyclinD1, PCNA, MMP‐7, and CD44v6. This panel was developed using tissue samples from patients with pancreatic cancer and was able to predict patient survival more accurately than individual marker alone [53].
2. Panel includes Ki‐67, cyclinD1, p53, and p16. This panel was developed using tissue samples from patients with pancreatic cancer and was able to predict patient survival and disease progression more accurately than any single marker alone [53].
3. Panel includes genomic and imaging data, as well as clinical information, and was developed using machine learning algorithms. This panel incorporated multiple biomarkers, including Ki‐67 and PCNA, radiomic features and clinical variables, and was able to accurately predict patient survival and response to treatment [54].
4. Panel includes circulating tumor cells (CTCs), CA19‐9, and C‐reactive protein (CRP). This panel was developed using blood samples from patients with pancreatic cancer and was able to predict patient survival and treatment response more accurately than any individual marker alone [55, 56].

Mainly, these multi‐marker panels show promise in improving the accuracy of prognostic and predictive modeling in pancreatic cancer. However, further research is needed to validate these panels and to determine the most effective ways to incorporate them into clinical practice. The use of multi‐marker panels in pancreatic cancer diagnosis has several potential benefits, including:

1. **Improved accuracy:** Multi‐marker panels can provide a more comprehensive view of the molecular profile of pancreatic cancer and may improve the diagnosis accuracy. By incorporating multiple biomarkers, these panels can help to identify different subtypes of pancreatic cancer and provide more personalized treatment options [57].
2. **Early detection:** Multi‐marker panels may be able to detect pancreatic cancer at an earlier stage than conventional diagnostic methods, such as imaging tests or biopsies. This could lead to earlier treatment and improved patient outcomes [58].
3. **Prognostic information:** Multi‐marker panels can provide valuable prognostic information, such as the likelihood of disease progression and response to treatment. This can help clinicians to make more informed treatment decisions and improve patient outcomes [58].
4. **Treatment planning:** Multi‐marker panels can aid physicians in gauging patient responses to chemotherapy or the resectability of tumors [59].
5. **Monitoring disease progression:** Multi‐marker panels can be used to monitor disease progression and treatment response over time. This can help clinicians to adjust treatment as needed and improve patient outcomes by performing followup biopsies [60].
6. **Cost‐effectiveness:** By identifying the most effective treatment options for each patient, multi‐marker panels may ultimately lead to cost savings by minimizing the necessity for ineffective treatments and enhancing overall patient outcomes [61].

In general, the use of multi‐marker panels in pancreatic cancer diagnosis has the potential to improve accuracy, enable earlier detection, provide valuable prognostic information, monitor disease progression, and improve cost‐effectiveness. Further research is needed to validate the clinical utility of these panels and to determine the most effective ways to incorporate them into clinical practice [62].

### 3.3 The Prognostic Value of the Markers

In contemporary oncology practice, most patients with pancreatic cancer undergo neoadjuvant chemotherapy, with most frequent regimens of FOLFIRINOX or gemcitabine/nab‐paclitaxel. Neoadjuvant chemotherapy should be considered preceding surgical resection due to the potential effect to increase the possibility of margin‐negative resection [63]. A study described a computational model to explore the metastatic aspect of pancreatic cancer and demonstrated that targeting cell proliferation with chemotherapy is more appropriate than surgery [64]. However, a remarkable proportion of cases develop resistance to chemotherapy. Novel therapies are required to prevent the signaling pathways from developing chemotherapeutic resistance. The complicated genetic changes in pancreas cancer could be used to plan new individualized treatment strategies. A common pancreatic tumor is molecularly heterogeneous and accommodates approximately 63 genomic modifications and variations [65].

**Figure 2:**
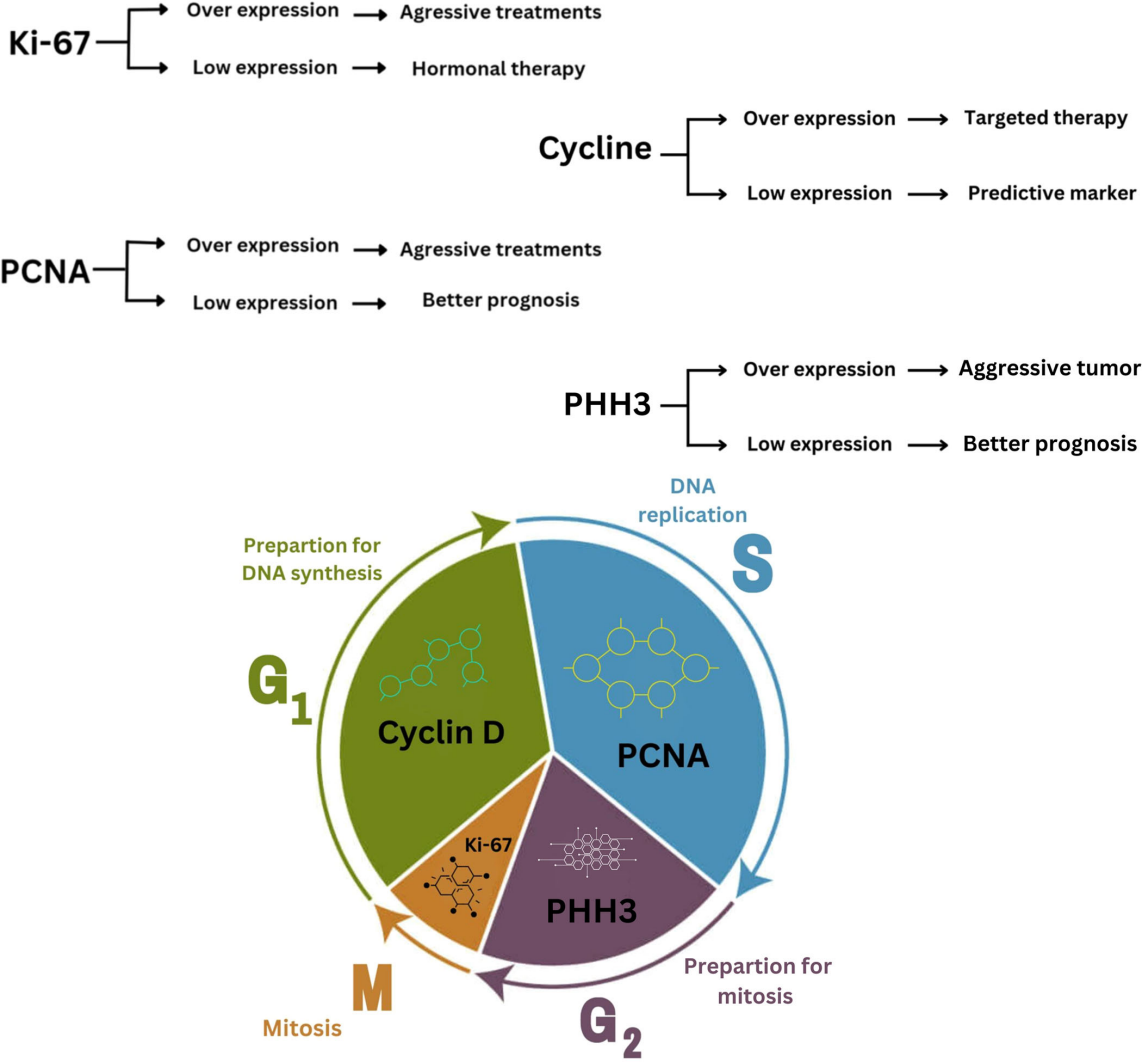
Role of marker expression in clinical management of pancreatic cancer and timing in the cell proliferation cycle. This figure illustrates the impact of overexpression and low expression of key proliferative markers (Ki‐67, Cyclin D1, PHH3, and PCNA) on the clinical management of pancreatic cancer and highlights their production timing during the cell proliferation cycle.

Thus, new biomarkers are required to predict survival or even indicate the effectiveness of surgery and other treatment modalities. Biomarkers could offer individual therapeutic approaches based on the personal profile of the genome and clinicopathology [66]. These tumor biomarkers not only aid in predicting the specific patient groups likely to benefit from chemotherapy but also play a crucial role in tailoring individualized therapy based on the unique characteristics of the patient's tumor [67]. Figure 2 summarize the role of marker expression in clinical management of pancreatic cancer and timing in the cell proliferation cycle.

#### 3.3.1 Cyclin

The cell cycle consists of four subsequent phases, gap 1 (G1), synthesis (S), gap 2 (G2), and mitosis (M), respectively, which are closely controlled by cyclin/cyclin‐dependent kinase (CDK) complexes. Cell growth is mainly held in the G1 phase, and the factors regulating entrance from G0 through G1 are essential for evaluating overall growth rates [68]. CDK complexes manage to progress through the cell cycles by activation and phosphorylation. Each cyclin indicates a particular pattern of expression and degradation. Cyclins D and E, which have been known as G1 cyclins, bind to CDK4 or six and CDK2, phosphorylate the retinoblastoma protein (Rb), simplifying the transition from G1 to S phase, which indicate the critical role in cell‐cycle regulation [69], resulting in neoplasm growth and malignancy progression. Hyperphosphorylation of Rb leads to the release of E2F transcription factors, which are needed to express the S phase genes [70].

Deregulation of cyclin D1 can cause genetic tumorigenesis instability [71]. Misrepresentation of the CCND1 gene encoding cyclin D1 and overexpression of cyclin D1 protein have commonly been found in various malignant neoplasms [72, 73, 74]. Therefore, cyclin D1 has become known for its remarkable role in guiding tumorigenesis. Furthermore, overexpression of cyclin D1 mRNA is associated with a decreased survival rate in pancreatic cancer, similar to its immunostaining results [31, 75]. Moreover, the overexpression of cyclin D1 in pancreatic cancer is correlated with a reduced postoperative survival rate [31, 76]. Nuclear overexpression of cyclin D1 protein was observed by immunohistochemistry in about 60% to 85% of invasive pancreatic adenocarcinoma [77]. In previous reports, the frequency of overexpression of D1 cyclin in solid pseudopapillary tumors attained 70‐100% [78, 79, 80, 81, 82]. Cells overexpressing cyclin D1 are susceptible to poor prognosis and resistance to chemotherapy, especially in pancreatic cancer [83].

#### 3.3.2 Ki‐67

Ki‐67 is a nuclear protein expressed in all cell cycle phases except G0, with the highest expression rate observed in the G2 and M phases [84]. The expression of ki‐67 is characteristic of tumor proliferation and is associated with progression, metastasis, and prognosis [85]. Previous studies demonstrated the association between increasing Ki‐67 expression and malignancy progression in pancreas cancer [86]. Several studies and guidelines have shown that the Ki‐67 value could be a valid biomarker, and it could substitute for clinical course observation to display the indication of chemotherapy. On multivariate analysis, ki‐67 remained significant in disease progression and implied that every 1% unit increase in ki‐67 leads to a 3% increase in the progression risk [67].

Most studies suggest that the Ki‐67 index is a valuable prognostic indicator in pancreas neuroendocrine tumors. Since the 2010 WHO classification, the grading of pancreatic neuroendocrine tumors has been mainly based on the Ki‐67 index. In 2004 the Ki‐67 index became a crucial prognostic factor for the classification of pancreatic neuroendocrine neoplasms (PanNENs) and has been demonstrated in the protocols and guidelines applied by the North American Neuroendocrine Tumor Society [87], American Joint Committee on Cancer (AJCC) [88] and European Neuroendocrine Tumor Society [89]. Additionally, results have demonstrated that the Ki‐67 index is the most valuable factor in the prognostic assessment of PanNENs since small biopsy specimens contain inadequate tissue to evaluate mitotic count in 10HPF [90].

In another study, they suggest a novel categorization of the low‐grade and intermediate‐grade pancreatic neuroendocrine tumors based on the expression of Ki‐67 to predict recurrence after curative surgery. The risk of recurrence of pancreas tumors with Ki‐67 6–20% is threefold higher than those with Ki‐67 < 5% within 5 years and significantly shorter survival [91]. The Ki‐67 labeling index has been applied as a proliferative marker due to a measurable and repeatable method to evaluate the growth rate of normal and malignant cells. Ki‐67 immunostaining procedures have been standardized by automated staining machines. However, there are still differences between laboratories due to variability in tissue processing and fixation [92]. It has been observed that Ki‐67 staining of core biopsies usually prepare a reliable method of proliferation evaluation for the prognosis of metastatic liver NETs [93].

#### 3.3.3 Proliferating Cell Nuclear Antigen

Overexpression of PCNA was related to various gastroinestinal system cancers [94, 95]. One study extracted RNA sequencing data from G‐TEx (Genotype‐Tissue Expression) and TCGA (The Cancer Genome Atlas) databases to establish relative PCNA expression in PDAC and normal pancreas. The result indicated that PCNA is remarkably overexpressed in pancreatic cancers compared to normal pancreas. Additionally, pancreatic cancer cells showing PCNA expression are related to poor prognosis [96].

Furthermore, analysis using the Kaplan‐Meier Plotter demonstrated that increased PCNA expression was associated with poor survival in the TCGA cohort. The median overall survival for the PCNA‐low cohort was 35.3 months, while the PCNA‐high cohort was 17.3 months. These analyses showed that enhanced expression of PCNA is associated with worse survival [96]. Not only PCNA protein expression rate was higher in the moderate differentiated group than that in the well‐differentiated, but also PCNA protein expression rate was higher in patients with positive lymph node than in the lymph node‐negative patients. These findings suggested that determining the malignant proliferating status of pancreatic cancer by expression of PCNA may be a practical value, and overexpression of PCNA protein may indicate the progression of pancreatic cancer [94].

#### 3.3.4 Phosphorylated Histone H3

The anti‐PHH3 antibody addresses several limitations in conventional staining methods. Specifically targeting phosphorylated H3, a protein uniquely present during the M phase, it eliminates the ambiguity associated with H&E scoring [97]. Moreover, PHH3 exhibits vivid staining of cells in mitosis while avoiding apoptotic cells. Its heightened specificity and clear visual contrast between positive and negative cells make mitotic count readings with PHH3 both simpler and less time‐consuming compared to standard H&E staining [98]. Both PHH3 and H&E evaluations were completed within approximately 3 min. However, the predictive value of PHH3 surpassed that of H&E staining. In contrast, Ki‐67 counts required an average of 15 min, yet with a predictive value inferior to that of PHH3 [99, 100].

Beyond its enhanced specificity for staining mitotic cells, the PHH3 mitotic rate count demonstrates low interobserver variation, as evidenced by numerous studies, including recent research on Pancreatic Neuroendocrine Tumors reporting almost perfect (*k* > 0.98) interobserver agreement. Significantly, due to its high specificity for mitoses, PHH3 provides a more accurate reflection of the tumor's biology, likely forming the basis for its superior performance in predicting patient survival [101, 102].

Moreover, PHH3 proves valuable in predicting treatment response. A high mitotic index, indicated by PHH3 expression, may suggest a more proliferative and potentially chemotherapy‐resistant tumor [103]. This information becomes pivotal in tailoring treatment strategies, enabling clinicians to choose more aggressive therapeutic approaches for patients with tumors exhibiting heightened mitotic activity [104]. The multifaceted advantages of PHH3 underscore its potential as a powerful tool in both diagnostic and prognostic assessments for pancreatic cancer [105].

### 3.4 The Effect of Chemotherapy on the Markers

Recent advancements have witnessed improvements in targeted molecular therapies aimed at inhibiting specific pathways [106]. Furthermore, contemporary approaches involve combination therapies, pairing these agents with conventional treatments like gemcitabine [106]. These combinations are designed to enhance effectiveness through synergistic effects, enabling the use of lower doses for individual agents and reducing overall toxicity [107].

Relevent to this, a new drug of parthenolide with enhanced bioavailability, called LC‐1 (also known as DMAPT or dimethylamino‐parthenolide), was manufactured. A study examining the effects of sulindac in pancreatic cancer treatment revealed that high‐dose sulindac alone and low‐dose combined with LC‐1 effectively suppressed pancreatic tumor expansion. Moreover, these treatments were associated with a decrease in the cell cycle regulatory protein cyclin D1 and reduction in Ki‐67 staining resulting in inhibition of proliferation [107]. Therefore, the observed decrease in tumor size following LC‐1/sulindac combination or high‐dose sulindac treatments may be attributed to the prevention of cyclin D1 production, causing significant chemosensitivity [107].

Suppression of cyclin D1 expression by cyclin D1 antisense prevented cell growth and tumorigenicity [83]. In addition, a study previously reported the reduction of cyclin D1 expression following therapy of pancreatic cancer cells with nonsteroidal anti‐inflammatory drugs such as sulindac, which suppress cell cycle progression [108]. Cyclin D1 suppression levels can enhance the response of pancreatic cancer cells to cisplatinum [83]. Also, suppression of cyclin D1 by small interfering RNA increased sensitivity to apoptosis by cisplatin [109]. Overexpression of c‐myc in pancreatic cancer caused enhancement of sensitivity to cisplatin‐induced cell death due to inhibition of cyclin D1 expression [110]. Moreover, CCND1 has been tested as a therapeutic target by cyclin‐dependent kinase inhibitors in ductal adenocarcinoma of the pancreas. However, further evaluations of the diagnostic and therapeutic role of cyclin D1 are suggested [66].

Additionally, overexpression of cyclin D1 can lead to resistance of pancreatic cancer to cytotoxic drugs chemotherapy [74, 109, 111, 112]. Evidence also hypothesized that tumors with higher Ki‐67 may respond better to chemotherapy; however, this should be tested in more prospective clinical studies [113]. Results of a recent study indicated that Ki‐67 values had no effect on overall survival, though, in patients with a higher Ki‐67 proliferation index, the progression‐free survival was remarkably poorer. However, this negative influence was only observed in the Gemcitabine‐based treated group, not in the FOLFIRINOX group. Accordingly, this study revealed that the gemcitabine‐based could be effective in patients with lower Ki‐67 and p53 values. However, the FOLFIRINOX regimen chemotherapy has been superior to gemcitabine regimen chemotherapy in metastatic pancreatic cancer [114]. The Ki‐67 is easily measured in clinical care and hence could serve as prognostic markers for metastatic cancer tumors, likely to respond to gemcitabine chemotherapy instead of FOLFIRINOX [67]. Further evaluations are necessary to assess the chemotherapeutic effect of drugs on patients due to individual state of biomarker indexes.

## 4 Comparative of Proliferative Markers and Traditional Biomarkers

Proliferative markers (Ki‐67, PCNA, Cyclin D1, PHH3) serve as valuable indicators of pancreatic cancer behavior and treatment response [115]. High Ki‐67 expression correlates with increased tumor aggressiveness and poor survival, while PCNA and PHH3 indicate DNA replication and mitotic activity [116]. These markers can guide therapeutic decisions by predicting chemotherapy response, enabling treatment personalization [117]. Traditional markers like CA19‐9, while widely used for their noninvasive nature, have limited specificity and may be elevated in benign conditions. Furthermore, Lewis antigen‐negative patients cannot produce CA19‐9 [115]. Though proliferative markers provide detailed tumor insights, they require tissue sampling and face standardization challenges [116]. Emerging liquid biopsy markers (circulating tumor DNA, microRNAs, exosomes) offer promising noninvasive early detection methods but require further validation [118]. An integrated approach combining proliferative markers' detailed tumor characterization with traditional and emerging serum‐based markers' convenience could optimize patient monitoring and treatment outcomes [116]. Table 3 comprises proliferative markers and traditional biomarkers in pancreatic cancer.

**Table 3:** Comparative table of proliferative markers and traditional biomarkers in pancreatic cancer.

Biomarker	Type	Clinical utility	Sensitivity	Specificity	Key limitations
Ki‐67	Proliferative	Prognostic, indicates tumor aggressiveness	Moderate	Moderate	Lack of standardization in cut‐off values, variability in tumor regions
PCNA		Marker for DNA replication and repair	High	Low	Overexpressed in various cancers, not specific to pancreatic cancer
Cyclin D1		Prognostic, linked to cell cycle progression	Moderate	Moderate	Limited validation in clinical trials, lacks established therapeutic guidance
CA19‐9	Traditional	Diagnostic and monitoring tool	80%	75%	Elevated in benign conditions (pancreatitis), not expressed in 10% of population
CEA		Diagnostic, used in conjunction with CA19‐9	Low	Low	Inconsistent results, lacks specificity for pancreatic cancer
CA125		Diagnostic and prognostic tool	59%	78%	Limited by small study sizes, may not be superior to CA19‐9 in isolation

### 4.1 Future Direction: Digital Pathology

Artificial intelligence (AI) and machine learning have emerged as transformative solutions for improving the utility of biomarkers in pancreatic cancer, particularly in addressing challenges related to interobserver variability and standardizing time‐consuming tasks. One promising application of AI in this field is digital pathology, which involves the digitization of pathology slides, enabling advanced technologies such as machine learning to assist in pattern recognition and analysis [119]. This innovation can significantly enhance the accuracy and efficiency of pathological assessments, leading to more precise diagnoses and ultimately improving patient careple, automated Ki‐67 scoring using image analysis of whole slide images has been developed to standardize the reporting of Ki‐67, which has historically suffered from variability in assessment [120]. This automation effectively addresses previous barriers related to inconsistency in Ki‐67 evaluations and the time constraints of manual counting. In pancr adenocarcinoma (PDAC), there has been disagreement regarding the best approach to count Ki‐67, with some advocating for counting in “hot spots” of high proliferative activity and others supporting an average count across the tumor section. This lack of uniforeded the development of globally recognized standards for evaluating PDAC proliferation [121].

Automated Ki‐67 counting addresses standardization challenges by providing objective scoring across tumor regions, including hot spots and leading edges, while capturing geographic heterogeneity in proliferative activity(130,132). AI applications in pancreatic pathology have demonstrated impressive capabilities, achieving 93% accuracy in PDAC diagnosis and precise perineural invasion segmentation (133). These technologies promise integrated analysis tools combining pathology images, biomarker data, clinical information, and radiological findings (134–136).

However, digital pathology in pancreatic cancer remains nascent [122]. While image processing tools show promise in preclinical models, advancement requires additional research and comprehensive data registries (137138, 139, 140). The integration of big data and machine learning offers potential for merged analysis of multiple data sources, approximating expert clinical assessment (141).

## 5 Discussion

Pancreatic cancer is one of the most aggressive malignancies with a low survival rate, and chemotherapy is a standard treatment option for pancreatic cancer [123]. However, the response to chemotherapy varies significantly among patients, highlighting the crucial need for reliable biomarkerd to predict the efficacy of chemotherapy and personalize treatment strategies [124].

Proliferative markers, such as Ki‐67, PCNA, PHH3, and Cyclin D1, have been investigated for their potential use as predictive and prognostic biomarkers in pancreatic cancer chemotherapy. Ki‐67 is a well‐established marker of proliferation, and its expression is associated with tumor aggressiveness [38]. A reduction in Ki‐67 levels after chemotherapy is associated with a better response to treatment and improved survival outcomes. However, the use of Ki‐67 as a biomarker has its own limitations, including the absence of widely recognized threshold and the lack of specificity for tumor cells [90].

PCNA is involved in DNA repair and can differentiate between proliferating and non‐proliferating cells. Its expression is induced by growth factors or in response to damaged DNA, even after the cell is no longer active in the cell cycle. PCNA has been associated with poor outcomes in pancreatic cancer, and studies have suggested that its phosphorylation status may be a potential biomarker for predicting chemotherapy response [125].

PHH3, as a mitotic marker, plays a crucial role in identifying cells actively engaged in the late stages of the cell cycle (G2 and M phases). In pancreatic cancer, where the proliferative activity often correlates with disease aggressiveness, the assessment of mitotic activity using PHH3 becomes particularly relevant [29].

Cyclin D1 is a cell cycle regulatory protein that plays a critical role in G1‐S phase transition. Its overexpression has been associated with tumor growth and poor prognosis in pancreatic cancer. Cyclin D1 has also been investigated as a potential biomarker for predicting chemotherapy response in pancreatic cancer. Studies have suggested that high Cyclin D1 expression may be associated with resistance to chemotherapy and poor outcomes [126].

Despite the positive results linked to these proliferative markers, their smooth integration into clinical practice poses significant challenges. Essential to this endeavor is the standardization of techniques and scoring methodologies, a critical step towards incorporating these markers into routine clinical practice [122]. Furthermore, a comprehensive comprehension of the intricate interplay between these markers and various molecular and clinical factors that intricately influence chemotherapy response is imperative [127]. Embracing digital pathology may offer solutions to some of these implementation barriers, notably by mitigating interobserver variability, revealing hitherto unforeseen clusters, and facilitating individualized treatment planning [128].

The incorporation of proliferative markers like Ki‐67, PCNA, and Cyclin D1 into treatment decision‐making for pancreatic cancer is promising but comes with significant limitations that restrict their current clinical utility. One of the most discussed markers, Ki‐67, has demonstrated a clear correlation with cell proliferation, with higher expression levels often associated with poorer outcomes and more aggressive disease. However, using Ki‐67 as a prognostic marker in pancreatic cancer is complicated due to conflicting results reported across various studies. Some studies, such as those by Klein et al. and Zińczuk et al., have shown a strong association between increased Ki‐67 labeling indices and higher grades of pancreatic intraepithelial neoplasia (PanIN) and invasive pancreatic ductal adenocarcinoma, suggesting that Ki‐67 could potentially be used to identify high‐risk patients.

However, other studies have not consistently replicated these findings, with some reporting that high Ki‐67 levels do not always correlate with poor survival outcomes. Several factors might explain this discrepancy, including differences in study design, patient populations, and methodologies used for assessing Ki‐67 expression. For instance, variability in tissue sample handling, differences in antibody clones used for immunohistochemistry, and variations in the definition of “high” or “low” Ki‐67 expression thresholds can all influence the results. Additionally, Ki‐67 expression might reflect short‐term proliferation rates that do not capture the full complexity of tumor biology, such as the potential for quiescent cells to become reactivated under certain conditions. These factors contribute to the challenges in standardizing Ki‐67 assessment and its interpretation, making it difficult to integrate into clinical decision‐making protocols without further research to establish consistent methodologies and clear cutoff values. Until such standardization is achieved, the role of Ki‐67 in guiding treatment decisions remains limited, and clinicians should use it alongside other clinical and pathological factors rather than as a standalone marker.

In conclusion, proliferative markers including Ki‐67, PCNA, PHH3 and Cyclin D1 have demonstrated promise as predictive and prognostic biomarkers in the context of pancreatic cancer chemotherapy. These markers offer the potential to forecast chemotherapy response and tailor treatment approaches to individual patients. However, the translation of these markers into routine clinical practice requires further comprehensive investigations and validation. The establishment of biomarkers holds the potential to significantly enhance the clinical outcomes and personalized care of individuals affected by pancreatic cancer.

## Author Contributions

**Aryan Salahi‐Niri:** conceptualization, literature review and writing the original draft, visualization. **Paniz Zarand:** literature review and writing the original draft. **Negar Mansouri:** literature review and writing the original draft. **Parvaneh Rastgou:** commenting and editing the manuscript. **Omid Yazdani:** commenting and editing the manuscript. **Romina Esbati:** commenting and editing the manuscript. **Fatemeh Shojaeian:** litreature review; commenting and editing the manuscript. **Behnaz Jahanbin:** conceptualization, validation, commenting and editing the manuscript. **Zhaleh Mohsenifar:** validation, commenting and editing the manuscript. **Hamid Asadzadeh Aghdaei:** validation; commenting and editing the manuscript. **Farid Azmoudeh Ardalan:** validation; commenting and editing the manuscript. **Seyed Amir Ahmad Safavi‐Naini:** conceptualization, literature review and writing the original draft, supervision, administration.

## Ethics Statement

The authors have nothing to report.

## Conflicts of Interest

The authors declare no conflicts of interest.

## Transparency Statement

The lead author Seyed Amir Ahmad Safavi‐Naini affirms that this manuscript is an honest, accurate, and transparent account of the study being reported; that no important aspects of the study have been omitted; and that any discrepancies from the study as planned (and, if relevant, registered) have been explained.

## Data Availability

All data and summarization are available within the manuscript and supplementary files.
